# DOES RECTUS FEMORIS TRANSFER INCREASE KNEE FLEXION DURING STANCE PHASE IN CEREBRAL PALSY?

**DOI:** 10.1590/1413-785220162401145765

**Published:** 2016

**Authors:** Mauro César de Morais, Francesco Camara Blumetti, Cátia Miyuki Kawamura, José Augusto Fernandes Lopes, Daniella Lins Neves, Michelle de Oliveira Cardoso

**Affiliations:** 1. Associação de Assistência à Criança Deficiente (AACD), Gait Laboratory, São Paulo, SP, Brazil.; 2. Universidade de São Paulo, Faculdade de Medicina, Hospital das Clínicas, Instituto de Medicina de Reabilitação (IMREA/HC/FMUSP), São Paulo, SP, Brazil.; 3. Universidade de São Paulo, Faculdade de Medicina, Hospital das Clínicas, Instituto de Ortopedia e Traumatologia, Paralysis Group, São Paulo, SP, Brazil.

**Keywords:** Knee joint, Gait, Range of motion, articular, Cerebral palsy

## Abstract

**Objective:**

To evaluate whether distal rectus femoris transfer (DRFT) is related to postoperative increase of knee flexion during the stance phase in cerebral palsy (CP).

**Methods:**

The inclusion criteria were Gross Motor Function Classification System (GMFCS) levels I-III, kinematic criteria for stiff-knee gait at baseline, and individuals who underwent orthopaedic surgery and had gait analyses performed before and after intervention. The patients included were divided into the following two groups: NO-DRFT (133 patients), which included patients who underwent orthopaedic surgery without DRFT, and DRFT (83 patients), which included patients who underwent orthopaedic surgery that included DRFT. The primary outcome was to evaluate in each group if minimum knee flexion in stance phase (FMJFA) changed after treatment.

**Results:**

The mean FMJFA increased from 13.19° to 16.74° (p=0.003) and from 10.60° to 14.80° (p=0.001) in Groups NO-DRFT and DRFT, respectively. The post-operative FMJFA was similar between groups NO-DRFT and DRFT (p=0.534). The increase of FMJFA during the second exam (from 13.01° to 22.51°) was higher among the GMFCS III patients in the DRFT group (p<0.001).

**Conclusion:**

In this study, DRFT did not generate additional increase of knee flexion during stance phase when compared to the control group. ***Level of Evidence III, Retrospective Comparative Study.***

## INTRODUCTION

Stiff knee gait is a frequent problem for people with cerebral palsy (CP) and it is characterized by a reduction in knee flexion during the swing phase.^1^ Rectus femoris (RF) spasticity, poor pre-swing ankle power generation, decreased walking velocity and hip flexor weakness have been described as possible causes of stiff knee during CP.^2^

Distal rectus femoris transfer (DRFT), as described by Perry,^3^ is a well-established procedure to treat stiff knee gait that is related to RF spasticity. Most of the DRFT literature demonstrates improvements in knee range of motion after surgery; however, few studies have mentioned poor outcomes related to residual knee flexion during the stance phase.^4^ ^-^ ^13^

On the other hand, one of the findings in crouch gait is the increase of knee flexion during all stance phase, greater than 30°.^1^ Although the increase of ankle dorsiflexion and hip flexion during stance phase are problems frequently associated to crouch gait, Sutherland and Davids^1^ described the increase of knee flexion as a key point in the definition of this pattern. 

An increase in knee flexion during stance phase after DRFT was reported previously.^9^ ^,^ ^10^ Additionally, Gage et al.^1^ related the poorest post-DRFT outcomes to residual knee flexion during the stance phase and lever arm dysfunction. However, identifying the patients who are most susceptible to an increase in knee flexion during stance phase after DRFT is still a point of debate.

The primary purpose of this study was to analyse if DRFT produces an increase of knee flexion during stance after surgery. The secondary objective was to evaluate if the changes in stance phase after DRFT were similar among GMFCS levels I, II and III.

## MATERIALS AND METHODS

A retrospective cohort study was performed in a tertiary hospital and rehabilitation centre, and the local ethics committee approved the study (protocol 23/2013). The free informed consent form was obtained from each patient during data collection at gait laboratory. 

A search of the gait laboratory database was conducted, and the inclusion criteria were as follows: (1) diagnosis of diplegic spastic CP; (2) GMFCS from I to III; (3) maximum peak knee flexion during stance phase < 50°; (4) indication for DRFT in gait laboratory report; and (5) patients who underwent lower limb orthopaedic surgery and had gait analyses conducted on them before and after the intervention (12 months or more after surgery). We excluded patients with incomplete documentation at the gait laboratory (pre-operative gait analyses done more than 12 months before surgical intervention) and those who underwent other rectus femoris procedures (proximal release, intramuscular lengthening, distal tenotomy and proximal transfer). 

To collect the kinematic data, reflective markers were strategically placed on specific anatomical landmarks on the participants, as described by Kadaba et al.^14^ The trajectory of the markers within the lab space was captured through an electronic optical system that consisted of infrared cameras. Until August 2008, a 6-camera Vicon 370 system 60 Hz (UK) was used for data capture; however, from this date on, an 8-camera Qualisys Oqus300 system 500 Hz (Sweden) was used.

The patients were instructed to walk barefoot at a self-selected speed in an 8-metre walkway (26 feet). A minimum of six gait cycles for each assessed lower limb were collected for consistency evaluation. The data were processed using the Vicon Clinical Manager software (VCM, Oxford Metrics, Oxford, UK) according to the technique described by Davis et al.^15^

According inclusion criteria, all of studied patients had indication for DRFT in three dimensional gait analyses. Nevertheless, the gait analysis report is just one of parameters used to define the final treatment plan. Despite the fact that DRFT had been suggested by gait analyses, many surgeons decided to perform this procedure later, after the correction of knee flexion deformity and lever arm dysfunction, for instance. Because this, it was created a convenient opportunity to use patients those did not received the DRFT as a control group. Finally, Paediatric orthopaedic surgeon's staff of our hospital, following the same surgical technique and post-operative protocol, performed all of the procedures.

To check if the groups were matched, their ages at surgery, gender distribution, GMFCS levels,^16^ surgeries performed, follow-up times, pre-operative Gait Deviation Indexes (GDI)^17^ and pre-operative minimum knee flexion levels in the stance phase were analysed and the results were compared. The primary outcome was changes in minimum knee flexion in stance phase (MKFSt) following treatment in each group, and the secondary outcome was the correlation of it with GMFCS levels. Additionally, the peak knee flexion in swing phase (PKFLSw) and knee range of motion (KRM) were analysed before and after the surgical procedures in both groups. 

A statistical analysis was applied with a 5% significance level. For the continuous variables, the paired Student's t test was applied, and the Two Proportions Equality and Chi Square Tests was used for categorical variables. 

## RESULTS

From the 4393 patients with CP observed in our gait laboratory from 1996 to 2013, 3283 of them were spastic diplegic and 410 fulfilled the inclusion criteria. One hundred ninety four subjects were excluded because they had incomplete documentation at the gait laboratory or they had received other rectus femoris procedures instead of DRFT. The remaining 216 patients (308 lower limbs) were divided into two groups: Group NO-DRFT (133 patients/185 knees) included patients who underwent single event multilevel orthopaedic surgery (SEMLS) without a DRFT, and Group DRFT (83 patients / 123 knees) included patients who underwent SEMLS that included a DRFT. In the Group NO-DRFT, 52 patients received bilateral procedures and 81 unilateral, while Group DRFT had 40 patients with bilateral procedures and 43 with procedures applied at one side only. 

Demographic data was similar between groups regarding gender, follow-up time (Group NO-DRFT 3.4 years and Group DRFT 3.3 years) and GMFCS level distribution. The mean age at the time of surgery was found to be higher for patients in the DRFT group (p=0.001). In the NO-DRFT group the age at surgery was 10.33 years, while in the DRFT group it was 12.6 years. Baseline kinematic data was similar between groups, except for PKFLSw, which was lower in the DRFT group (p=0.006). (Table 1)

**Table 1:** Demographic and baseline data from the included patients in each group.

Group no-DRFT		Group DRFT	
N	%	N	%	P-value
Total	185	100%	123	100
Gender
Male	119	64.3%	74	60.2%	0.46
Female	66	35.7%	49	39.8%	
GMFCS
Level I	15	8.1%	16	13%	0.262
Level II	101	54.6%	58	47.2%	
Level III	69	37.3%	49	39.8%	
Mean	SD	Mean	SD
Age (y)	10.33	5.61	12.60	5.82	0.001
Follow-up time (y)	3.40	2.74	3.38	3.19	0.953
Gait Analysis Parameters
GDI	54.66	12.16	56.82	11.89	0.125
MKFSt	13.19	14.49	10.6	14.26	0.123
PKFSw	46.30	6.69	43.38	8.45	0.006

DRFT (Distal Rectus Femoris Transfer), GMFCS (Gross Motor Function Classification System), SD (standard deviation), y (years), GDI (Gait Deviation Index), MKFSt (minimum knee flexion in stance), PKFSw (peak knee flexion in swing).

All patients of both groups received SEMLS after baseline gait analysis. Medial hamstring surgical lengthening and psoas lengthening over the pelvic brim were more prevalent in DRFT Group while triceps surae surgical lengthening was done more frequently in patients from NO-DRFT Group. The prevalence of other surgical procedures was not different between the groups. (Table 2) 

**Table 2:** Surgical procedures conducted for Groups no-DRFT and DRFT.

Surgical Procedures	Group no-DRFT		Group DRFT		P-value
	N	%	N	%	
HIP AD	142	76.8%	87	70.7%	0.236
PT	2	1.1%	0	0.0%	0.247
LAT HAM	8	4.3%	11	8.9%	0.099
MED HAM	128	69.2%	99	80.5%	0.027
FDO	64	34.6%	34	27.6%	0.199
FEO	12	6.5%	6	4.9%	0.556
POB	53	28.6%	54	43.9%	0.006
SPLATT	19	10.2%	16	13%	0.567
PV	38	20.5%	32	26%	0.567
TS	84	45.4%	35	28.4%	0.005

DRFT (Distal Rectus Femoris Transfer), HIP AD (hip adductors), PT (patellar tendon shortening), LAT HAM (biceps femoris surgical lengthening), MED HAM (medial hamstrings surgical lengthening), FDO (femoral derrotational osteotomy), FEO (femur extension osteotomy), POB (psoas lengthening over the pelvic brim), SPLATT (split of anterior tibialis tendon), PV (foot osteotomies for planus valgus correction) and TS (triceps surae surgical lengthening).

The MKFLSt was similar in both groups before and after the surgical interventions. There was an MKFLSt increase in Groups NO-DRFT and DRFT in the second gait analysis. Specifically, in Group NO-DRFT, MKFLSt increased from 13.19° to 16.74° (*p*=0.003), while in Group DRFT, MKFLSt increased from 10.60° to 14.80° (*p*=0.001). (Tables 3 and ^4^)

**Table 3:** Comparison of the minimum knee flexion at stance phase between Groups NO-DRFT and DRFT, before and after treatment.

MKFLSt	Group no-DRFT		Group DRFT	
	Pre-op	Post-op	Pre-op	Post-op
Mean	13.19˚	16.74˚	10.60˚	14.80˚
Median	13.88˚	15.84˚	11.70˚	12.70˚
SD	14.49˚	15.71˚	14.26˚	16.90˚
p-value	0.003		0.001	

DRFT (Distal Rectus Femoris Transfer), MKFLSt (minimum knee flexion at stance phase), SD (standard deviation).

**Table 4:** Comparison of minimum knee flexion at stance phase before and after treatment in Groups NO-DRFT and DRFT.

MKFLSt	Pre operative		Post Operative	
	no-DRFT	DRFT	no-DRFT	DRFT
Mean	13.19˚	10.60˚	16.74˚	14.80˚
Median	13.88˚	11.70˚	15.84˚	12.70˚
SD	14.49˚	14.26˚	15.71˚	16.90˚
p-value	0.123		0.302	

DRFT (Distal Rectus Femoris Transfer), MKFLSt (minimum knee flexion at stance phase), SD (standard deviation), Pre-op (pre-operative) and Post-op (post-operative).

The peak knee flexion in swing phase (PKFLSw) was lower in Group DRFT (43.38°) than in Group NO-DRFT (46.30°) during the pre-operative gait study (*p*=0.006); however, no differences were observed following treatment (*p*=0.117). An improvement in PKFLSw was noted in both groups during the follow-up evaluation. In Group NO-DRFT, the PKFLSw increased from 46.30° to 49.32° (*p*<0.001), while in Group DRFT, the PKFLSw increased from 43.38° to 51.43° (*p<*0.001). (Tables 5 and ^6^)

**Table 5:** Comparison of peak knee flexion at swing phase between Groups NO-DRFT and DRFT, before and after treatment.

PKFLSw	Group no-DRFT		Group DRFT	
	Pre-op	Post-op	Pre-op	Post-op
Mean	46.30˚	49.32˚	43.38˚	51.43˚
Median	47.79˚	50.36˚	43.88˚	52.45˚
SD	6.69˚	10.57˚	8.45˚	10.59˚
p-value	<0,001		<0,001	

DRFT (Distal Rectus Femoris Transfer), PKFLSw (peak knee flexion at swing phase), SD (standard deviation).

**Table 6:** Comparison of peak knee flexion at swing phase before and after treatment in Groups no-DRFT and DRFT.

PKFLSw	Pre operative		Post Operative	
	no-DRFT	DRFT	no-DRFT	DRFT
Mean	46.30˚	43.38˚	49.32˚	51.43˚
Median	47.79˚	43.88˚	50.36˚	52.45˚
SD	6.69˚	8.45˚	10.57˚	10.59˚
p-value	0.006		0.117	

DRFT (Distal Rectus Femoris Transfer), PKFLSw (peak knee flexion at swing phase), SD (standard deviation), Pre-op (pre-operative) and Post-op (post-operative).

The patients who received DRFT were stratified according to GMFCS levels, and it was possible to observe after stratification that MKFLSt only increased after treatment at level III patients (*p*<0.001). (Table 7) In addition to this, KRM increased at level I (from 33.7° to 41°, *p*=0.025) and II patients (from 36.1° to 42°, *p<*0.001). Patients GMFCS level III did not exhibit improvement at KRM (from 28.5° to 27.9°, *p*=0.751).

**Table 7:** Comparison of minimum knee flexion at stance phase before and after intervention between Groups NO-DRFT and DRFT, according to GMFCS distributions.

MKFLSt		Mean	Median	SD	p-value
GMFCS I	Group no-DRFT	Pre-op	7.38˚	11.43˚	20.21˚	0.176
		Post-op	13.04˚	14.84˚	9.29˚	
	Group DRFT	Pre-op	10.35˚	15.32˚	16.12˚	0.742
		Post-op	9.20˚	7.71˚	10.21˚	
GMFCS II	Group no-DRFT	Pre-op	10.25˚	10.70˚	12.52˚	0.002
		Post-op	14.62˚	14.95˚	13.58˚	
	Group DRFT	Pre-op	7.95˚	9.61˚	11.58˚	0.243
		Post-op	9.92˚	11.81˚	13.62˚	
GMFCS III	Group no-DRFT	Pre-op	18.57˚	18.69˚	14.49˚	0.225
		Post-op	21.44˚	20.25˚	18.77˚	
	Group DRFT	Pre-op	13.01˚	14.46˚	16.32˚	<0.001
Post-op	22.51˚	21.31˚	19.75˚

DRFT (Distal Rectus Femoris Transfer), MKFLSt (minimum knee flexion at stance phase), SD (standard deviation), Pre-op (pre-operative) and Post-op (post-operative).

The MKFLSt after surgical intervention was higher in patients who received medial hamstring surgical lengthening (MHSL) than those who did not. In Group NO-DRFT, the MKFLSt after treatment was 18.09° when MHSL was applied; however, it was only 13.72° for those who were not administered MHSL (*p*=0.0805). In Group DRFT, the MKFLSt was 16.02° and 8.09°, with and without MHSL, respectively (*p*=0.0297). 

Finally, we divided patients according to age at surgery in order to evaluate if this parameter had influenced the MKFLSt at final follow-up. In Group NO-DRFT, patients who received surgery before 10 years of age (mean 8.8 years), the MKFLSt at final follow up was 14.8° whereas at those with surgery at 10 years or older (mean 12.3 years), it was 19.4° (*p*=0.053). In Group DRFT, the MKFLSt was 18.8° before 10 years old (mean 9.1 years) and 12.8° for those 10 years or older (mean 12.8 years), respectively (*p*=0.066). 

## DISCUSSION

In the present study, we observed an increase in knee flexion in the stance phase in both groups during post-operative evaluations, and these results were similar when DRFT was or was not conducted. Additionally, the PKFLSw also improved in Groups NO-DRFT and DRFT. The MKFLSt increase was more significant in the GMFCS level III patients and the increase of KRM was noted only at GMFCS level II patients and I.

In 2002, Saw et al.^9^ described a 7.2° increase in knee flexion in the stance phase in a group of 18 patients who underwent a DRFT after a mean follow-up time of 4.6 years. The same amount of increase was noted by Carney et al.^10^ when DRFT was performed without concomitant hamstring lengthening. They studied 17 patients 12 months after DRFT, and nine of them were GMFCS level III. However, these studies did not use control groups for DRFT. All of patients received DRFT and it was not possible to compare the knee flexion in stance phase after surgical treatment in patients with and without DRFT.

In the present study, we observed an increase in knee flexion in the stance phase after treatment in both groups; however, this value was lower than values published by Saw et al.^9^ and Carney et al.^10^ According our data, MKFLSt was similar in groups NO-DRFT and DRFT before and after intervention.

In 2002, Bell et al.^18^ studied a group of patients with CP in order to evaluate the natural progression of gait after a mean follow-up time of 4.4 years. They observed that knee range of motion in sagittal plane and peak of knee flexion during swing phase had deteriorated over the time. In addition, knee extension during stance phase exhibited a trend to worsening as well. 

Considering all of this information together, we can state that the increase of MKFLSt observed at both groups during follow-up evaluation can be related to natural progression of gait in CP, as described by Bell et al.^18^

However, when the patients with DRFT were divided according to their GMFCS levels, it was noted that MKFLSt increased was not uniform. The GMFCS level III patients demonstrated the most significant rise in their knee flexion in the stance phase after a surgical intervention and presented the highest MKFLSt values during their follow-up analyses. 

These data suggest that GMFCS level III patients are more susceptible to an increase in knee flexion in the stance phase after DRFT. In 2006, Carney et al.^10^ observed an increase in knee flexion in the stance phase after DRFT, and the majority of their population was GMFCS level III. Moreover, in 2009, Rethlefsen et al.^8^ found that the poorest outcome after DRFT in their study was related to a loss of knee extension in stance phase, and this occurred in patients with limited ambulatory ability, mainly those who were GMFCS IV.

Additionally, Dreher et al.^19^ stated in 2012 that patients with flexed knee gait during the stance phase before surgery did not benefit from DRFT. The elimination of RF as a knee extensor may cause or aggravate an insufficiency in knee extension power. In the present study, GMFCS level III patients who received DRFT had 13.01° mean minimum knee flexion in the stance phase before surgery and this value was higher than observed at levels II patients and I.

Finally, we noted a peak knee flexion improvement in the swing phase of both groups. In 2012, Dreher et al.^19^ described a knee range of motion improvement during the swing phase in patients with and without DRFT as a part of multilevel orthopaedic surgeries, but the results were more remarkable in the DRFT patients. However, Dreher et al.^19^ observed a PKFLSw increase only in the patients who received DRFT in their study. In the present study, the PKFLSw was similar at both groups after surgery, but the group that received DRFT had lower swing phase flexion values during their pre-operative analyses. PKFLSw improvements were noted in both groups after orthopaedic surgery, but the amount of improvement was higher when DRFT was applied. In the present study, we observed that knee range of motion shown improvement after SEMLS only at patients GMFCS levels I and II, who received DFRT, which reinforces that more functional patients have better results after this procedure. 

The multifactorial aetiology of stiff knee gait and the participation of lever arm dysfunction can explain PKFLSw increases despite the inclusion of DRFT in the treatment plan. The simultaneous surgical correction of lower limb deformities can restore lever arms and consequently improve swing phase knee flexion values. In 1987, Gage et al.^11^ noted that excessive internal or external foot rotation that resulted in lever arm dysfunction was related to poor post-DRFT outcomes.

The number of patients studied and the fact that the groups were matched for gender distribution, follow-up time, pre-operative gait impairment and GMFCS distribution were strengths of this study. In contrast, the retrospective design, the higher MHSL and psoas lengthening over the pelvic brim (POB) prevalence in Group DRFT than in Group NO-DRFT and the fact that patients from group DRFT received surgical procedures at older age than Group NO-DRFT are limitations of the study. 

However, concomitant surgical hamstring lengthening was not related to better knee extension during stance phase after treatment. The MKFLSt after the surgical interventions was higher in the patients who received HSL than those who did not. In addition to this, the effect of POB at knee extension during gait has not been supported by current literature. The reduction of anterior pelvic tilt and pelvic range of motion at sagittal plane is described after POB, but the effect of it at hip and knee extension during stance phase remains unclear.^20^

About age at surgery, it is important to state that patients from Group DRFT received orthopaedic surgery older than Group NO-DRFT (10.3 years in Group A and 12.6 years in Group B). Deterioration of musculoskeletal deformities correction has more chance to occur after earlier interventions during growth and because of that, patients from Group NO-DRFT were potentially more susceptible to have an increase of knee flexion during stance phase than Group DRFT during follow-up. However, we did not observe significant difference at MKFLSt at both groups at final follow up between patients who received surgery before and after 10 years of age.

## CONCLUSION

In conclusion, DRFT did not generate additional increase of knee flexion during stance phase in the present study. Additionally, the post-surgery MKFSt increase was similar between Groups NO-DRFT and DRFT. Finally, GMFCS III patients who underwent multilevel surgery, including RFT, exhibited a higher increase in MKFSt after treatment than the GMFCS level I and II patients.
